# Patterns of Maternal and Paternal Adverse Childhood Experiences on Parenting Satisfaction and Parenting Stress

**DOI:** 10.1007/s10826-026-03351-9

**Published:** 2026-08-24

**Authors:** Linghua Jiang, Sara Vasilenko, Xiafei Wang

**Affiliations:** 1 Department of Human Development and Family Science, Syracuse University, Syracuse, USA; 2 College of Social Work, University of Kentucky, Lexington, USA; 3 Present Address: Institute for Behavioral Genetics, University of Colorado Boulder, Office 216, 1480 30th Street, CO 80303 Boulder, USA

**Keywords:** adverse childhood experiences (ACEs), latent class analysis (LCA), parenting stress, parenting satisfaction

## Abstract

Recent research has suggested that mothers who experienced a higher number of adverse childhood experiences (ACEs) may be more likely to have higher levels of parenting stress (Lange et al., 2019; Steele et al.,^2016^). However, few studies have examined fathers’ roles in the association. Also, it remains unclear how different combinations of maternal and paternal ACEs may affect parenting satisfaction and parenting stress. Using data from the National Longitudinal Study of Adolescent to Adult Health (Add Health; N = 2,578, 60.4% female), this study identified a five-class model for maternal ACEs: (1) Low ACEs (58.32%), Parental Substance Use and Divorce (20.22%), (3) Violence (10.52%), (4) Abuse (7.65%), and (5) Multiple ACEs (3.29%). For paternal ACEs, five-class models were identified: (1) Low ACEs (48.51%), (2) Parental Substance Use and Divorce (21%), (3) Violence Victimization (12.99%), (4) Household and Environmental Dysfunction (10.68%), and (5) Physical and Emotional Abuse (6.82%). LCA results revealed that parenting satisfaction differed across ACEs classes for both mothers and fathers, even though some ACEs classes might not differ from the Low ACEs class in the post-hoc tests. ACEs class memberships were not significantly associated with parenting stress at the omnibus level, although a pairwise comparison indicated higher stress in the Multiple ACEs class relative to the Low ACEs class. These findings suggest that prevention and intervention programs should target specific groups of parents exposed to different combinations of ACEs and address various aspects of parenting

## Highlights

Latent class analysis identified distinct patterns of ACEs among mothers and fathers.

ACE patterns were not significantly associated with parenting stress at the omnibus level for either mothers or fathers. In contrast, certain ACE patterns were associated with lower parenting satisfaction, with variations across gender.

Findings support gender-responsive, trauma-informed approaches in parenting interventions and policy.

Parenthood can be both challenging and rewarding in that it can be a potential source of stress, while also increasing life satisfaction and providing a sense of purpose (Henderson et al., 2016). Examining both parenting stress and parenting satisfaction is critical because these factors can affect parenting behavior and children’s developmental outcomes (Guajardo et al., 2009; Steele et al., 2016; Ward & Lee, 2020). Parenting stress can be defined as the psychological and physiological strain arising from the demands associated with the parenting role, often reflected in negative feelings and beliefs about oneself and the child (Deater-Deckard, 2008). Although related to broader psychological stress, parenting stress is conceptually distinct in that it reflects domain-specific challenges tied to parent–child interactions. In contrast, parenting satisfaction is a positive, subjective evaluation of the parenting role that emphasizes the rewarding and meaningful aspects of caregiving. Adverse childhood experiences (ACEs), i.e., stressful or traumatic events occurring before the age of 18, are associated with parenting stress and negative parenting practices (Hughes & Cossar, 2016; Lange et al., 2019; Steele et al., 2016). ACEs include child maltreatment, such as abuse and neglect, as well as household dysfunctions, including having family members with mental health issues, substance abuse within the household, witnessing domestic violence, having an incarcerated family member, and experiencing parental divorce or separation (Felitti et al., 1998).

However, while parenting stress has been the focus of several prior studies (Lange et al., 2019; Steele et al., 2016), parenting satisfaction, a positive, subjective dimension of the parenting experience has received little empirical attention in relation to ACEs. To our knowledge, no studies have directly examined how ACEs exposure relates to parenting satisfaction. Furthermore, it remains unclear how different combinations of ACEs, rather than cumulative scores, may differentially influence both parenting satisfaction and parenting stress, and how these associations may vary by parent gender. Understanding these nuances is crucial for identifying strategies to mitigate the intergenerational impact of adversity and promote more positive parenting experiences and child outcomes.

## Theoretical framework

Attachment theory suggests that humans are biologically predisposed to seek proximity and contact with a caregiver for comfort, security, and protection (Benoit, 2004; Bowlby, 1988; Bretherton, 1992). The internal working model is one of the core concepts of attachment theory that informs us to study the potential link between parents’ own ACEs and parenting experiences. Secure attachment to a primary caregiver fosters a positive internal working model where individuals see themselves as worthy, others as dependable, and relationships as trustworthy. Conversely, an insecure attachment experience shapes a model in which the self is perceived as unworthy, others as unresponsive, and relationships as unreliable (Bowlby, 1979; Shilkret & Shilkret, 2011). Individuals exposed to ACEs form insecure attachments during childhood (Baer & Martinez, 2006; Grady et al., 2017), while those insecure attachments may persist in adulthood (Grady et al., 2017) and influence their expectations, beliefs, and behaviors in subsequent social interactions (Hinnen et al., 2009). For example, compared with individuals who have experienced insecure attachments, those with secure attachments may perceive parenting as more enjoyable, less challenging, and less stressful.

Internal working models may manifest differently across genders. Women are more likely to develop interdependent self-construals that emphasize emotional connectedness and relational roles, whereas men are more likely to develop independent self-construals that prioritize autonomy and self-sufficiency (Cross & Madson, 1997). These gendered self-construals may shape how individuals internalize early attachment experiences, particularly in the context of ACEs, and subsequently influence their parenting. For example, mothers may be more susceptible to ACEs patterns involving disrupted caregiving relationships, as these experiences could directly affect both their internal working models and their relational orientation toward parenting. In contrast, although fathers with ACEs may also develop insecure attachments, their sense of role fulfillment may be more strongly affected by ACEs patterns, such as household instability or economic hardship, that undermine autonomy or goal-directed functioning, which in turn influence their parenting satisfaction or stress, particularly in areas such as providing for or guiding their children.

At the same time, because ACEs include a wide range of adversity types, different patterns of ACEs may differentially affect parenting outcomes. According to the Dimensional Model of Adversity (DMA; McLaughlin et al., 2014), childhood adversities can be distinguished by threat and deprivation dimensions. Threat refers to experiences involving direct harm or acute stress, such as serious physical injury, exposure to threatened death, or sexual violation, whereas deprivation refers to the absence of necessary cognitive, social, and environmental inputs essential for healthy child development, with neglect and poverty serving as prototypical examples (McLaughlin et al., 2014). Drawing on neuroscientific evidence, threat experiences are thought to primarily engage fear learning circuits centered in the amygdala and hippocampus, while deprivation is more likely to compromise prefrontal cortical development and the executive functions it supports (Sheridan & McLaughlin, 2014). Given that parenting satisfaction and parenting stress may each implicate distinct cognitive and emotional processes, it is important to examine how different patterns of ACEs may shape these parenting experiences to provide targeted family interventions.

## ACEs and Parenting

Since the seminal ACEs study by Felitti et al. (1998), a large body of research has documented the negative impact of ACEs on a range of physical and psychological health outcomes across the life course (Bellis et al., 2014; Hughes et al., 2017). More recently, increasing attention has been directed toward parenting as a key context through which the effects of parental ACEs may extend across generations. Evidence suggests that parental ACEs are associated with children’s internalizing and externalizing difficulties, partly through parenting-related factors such as parenting stress (Rowell & Neal-Barnett, 2022; Wang, 2022; Zhang et al., 2023). Accordingly, a growing body of research has examined the associations between parental ACEs and parenting experiences. Prior studies have shown that greater cumulative exposure to ACEs is associated with higher levels of harsh parenting and parenting stress (Lange et al., 2019; Morris et al., 2021; Steele et al., 2016). Specifically, parents experiencing childhood maltreatment tend to be more hostile toward their children, such as conducting more physical punishment and negative parenting practices like permissive and authoritarian parenting styles (Bailey et al. 2012; Plant et al. 2017; Zvara et al. 2017b). Parents’ sexual abuse history reduced the likelihood of spending time with the child and parental satisfaction (Ehrensaft et al., 2015).

Most ACEs research has relied on the cumulative risk (CR) approach that sums the number of different ACEs types to create a composite score of adversity exposure. This approach assumes that each ACE has an equivalent impact, which may overlook the heterogeneity of adversity experiences. For example, individuals with the same ACEs score may have different constellations of adversity, which can confer differential levels of risk (Barboza, 2018; Merians et al., 2019). Latent class analysis (LCA) emerges to overcome the limitation of the CR approach. With LCA, researchers can identify mutually exclusive subgroups of people who respond similarly to ACEs exposures, e.g., showing a similar pattern of ACEs constellation (Merians et al., 2019), thus to test how varying patterns of ACEs may have different associations with the study outcomes (Barboza, 2018; Wang et al., 2024). For example, one study (Lee et al., 2020) identified four classes of ACEs exposure among 10,784 young adults: Low Adversity (62.79%), Child Maltreatment (17.47%), Household Dysfunction (14.39%), and Community Violence (5.36%). The study found that compared to individuals in the Low Adversity class, the Child Maltreatment class was more likely to experience depression, anxiety and PTSD, the Community Violence class was more likely to report PTSD, while no significant differences were observed in the Household Dysfunction class in these three mental health outcomes in contrast to the Low Adversity class (Lee et al., 2020). Though LCA has been used to examine how different patterns of ACEs exposure predict health outcomes (Lee et al., 2020; Shin et al., 2018; Lacey et al., 2020), very few examined parenting outcomes.

This represents an important gap because different forms of adversity may have distinct implications for parenting experiences. For example, experiences characterized primarily by abuse may affect later parenting differently than experiences characterized by violence exposure, parental mental health problems or parental substance use and divorce, or experiences characterized by various combinations of abuse and household dysfunction. Examining ACEs as latent patterns rather than cumulative scores may therefore provide a more nuanced understanding of how specific constellations of adversity relate to parenting stress and parenting satisfaction.

Furthermore, prior studies on ACEs and parenting have predominantly focused on mothers (Lange et al., 2019; Madsen et al. 2022, Steele et al., 2016), leaving paternal ACEs underexplored. However, women and men may differ not only in the prevalence of specific types of ACEs, but also in how these experiences may shape parenting outcomes. A systematic review of 41 studies found that the prevalence of sexual abuse was consistently higher among women across most studies (Lee et al., 2026). In contrast, findings for physical and emotional abuse were mixed, with some studies reporting higher rates among women, others among men, and some showing no gender differences. Similarly, patterns for family mental health problems were mixed, although there was a general trend toward higher prevalence among women. Household substance abuse, dysfunction, and incarceration showed largely comparable prevalence across genders (Lee et al., 2026). These differences suggest that the constellations of ACEs may vary by gender, which in turn may differentially influence parenting experiences. In addition, gender differences in socialization and coping may shape how individuals respond to early adversity, potentially leading to variation in the associations between ACE patterns and parenting outcomes (Nolen-Hoeksema, 2012; Tamres et al., 2002). Therefore, the present study aims to address these research gaps by examining how ACEs patterns differ between mothers and fathers, and how these different patterns affect parenting satisfaction and parenting stress.

## The Present Study

Guided by attachment theory, the present study used latent class analysis (LCA) to identify distinct ACEs profiles among mothers and fathers using a large nationally representative sample of U.S. adults. We then examined how these ACEs classes were associated with both parenting satisfaction and parenting stress. Based on the findings from a scoping review on ACEs studies using LCA (Wang et al., 2024), we expected that distinct patterns of ACEs would be differentially associated with parenting satisfaction and parenting stress. For example, child maltreatment-related ACEs classes may be associated with more detrimental effects. According to existing research on gender differences in ACEs (Lee et al., 2026), we further expected that ACEs patterns would differ between mothers and fathers, and explored whether the associations between ACEs patterns and parenting outcomes vary by gender.

## Method

### Participants and Study Design

Data for the current study was drawn from the National Longitudinal Study of Adolescent to Adult Health (Add Health), which used a stratified and probability sampling to recruit adolescents from 7th grade to 12th grade from 132 middle and high schools in 1994 (more details, see Harris (2013)). To date, the Add Health study consists of five waves: WI (ages 12–18), WII (ages 13–19), WIII (ages 18–26), WIV (ages 24–32), and WV (ages 32–42). This study used data from the first four waves. In particular, ACEs were retrospective reports from WI, WIII and WIV, and the outcome variables parenting satisfaction and parenting stress were from WIV. The current study involved secondary analysis of archival Add Health data and was exempt from institutional review.

The study sample included 1,562 mothers (58.9% White, 11.4% Hispanic, 25.4% Black, 1.7% Asian, 2.6% other race; ages 25–34) and 1,016 fathers (60.3% White, 10.7% Hispanic, 25.4% Black, 1.6% Asian, 2.0% other race; ages 25–34). 71.6% of mothers and 72.8% of fathers experienced at least one ACE. The percentage of exposure to 4 and more than 4 ACEs is 5.2% for mothers, and 6% for fathers.

### Measures

#### Adverse Childhood Experiences (ACEs)

We constructed our ACEs measures primarily based on the 10-item from the original ACEs questionnaire (Felitti et al., 1998). Meanwhile, as researchers advocate for adding other ACEs indicators (Afifi, 2020; Choi et al., 2020; Cronholm et al., 2015), we also included five types of expanded ACEs: parental death, witnessing violence, violence victimization, foster care, and material hardship (Cronholm et al., 2015; Wang et al., 2024). At WI, WIII and WIV, participants retrospectively reported their ACEs happening before the age of 18. All ACEs items were coded as binary indicators. ACEs were constructed from items available in the Add Health dataset rather than derived from a standardized scale.

At WIII, physical abuse, physical neglect, and sexual abuse were respectively assessed by one question “By the time you started 6th grade, how often had your parents or other adult caregivers (a) slapped, hit, or kicked you? (b) not taken care of your basic needs, such as keeping you clean or providing food or clothing? (c) touched you in a sexual way, forced you to touch him or her in a sexual way, or forced you to have sexual relations?” Response categories include “1 one time”, “2 two times”, “3 three to five times”, “4 six to ten times”, “5 more than ten times”, and “6 This has never happened”. For physical abuse and neglect, the response was coded as 1 if participants reported more than six times, consistent with thresholds used in prior research (Assini-Meytin et al., 2022; Quinn et al., 2016). For sexual abuse, the response of at least one time was coded as 1. Otherwise, the responses were coded as 0.

At WIV, emotional abuse was measured by one question “Before your 18th birthday, how often did a parent or other adult caregiver say things that really hurt your feelings or made you feel like you were not wanted or loved?” The response categories were the same as those for physical abuse and neglect and were coded similarly (0 = never happened, 1 = more than six times). Emotional neglect was assessed using two items at WI, including “Most of the time, your mother/father is warm and loving toward you.” Response options ranged from 1 (strongly agree) to 5 (strongly disagree). Following prior research (Lee et al., 2020), responses of 4 (disagree) and 5 (strongly disagree) were coded as 1, and all other responses were coded as 0.

Parental separation or divorce was measured by one item at WI, “What is your current marital status?” (0 = married, 1 = otherwise). Parental conflict (WI) was assessed using the item, “How much do you fight or argue with your current (spouse/partner)?” Response categories included “1 = a lot,” “2 = some,” “3 = a little,” and “4 = not at all.” Responses of “a lot” were coded as 1 (high parental conflict), and all other responses were coded as 0 (low or no parental conflict). Parental incarceration (WIV) was assessed by whether biological mother/father, and mother/father figure “ever (spent/spend) time in jail or prison?” (0 = no, 1 = yes). Parental alcohol abuse (WI) was assessed using two indicators. First, parent report of whether the biological mother or father had alcoholism was used (0 = no, 1 = yes). Second, heavy alcohol use was measured using the item “How often in the last month have you had five or more drinks on one occasion?” Response categories were “1 never, 2 once, 3 twice, 4 three times, 5 four times, 6 five or more times.” Responses indicating at least one occasion were coded as 1, and “never” was coded as 0. Household suicidal attempt was assessed as having “any family members tried to kill themselves during the past 12 months?” (0 = no, 1 = yes).

The present study also included 5 types of ACEs as expanded ACEs: witnessing violence, violence victimization, parental death, foster care, and material hardship. All four expanded ACEs were assessed at WI, except for foster home experience, which was obtained from WIII.

Violence witnessing was assessed based on the frequency of the item “seeing someone shoot or stab another person during the past 12 months.” Response categories included 0 (never), 1 (at least once), and 2 (more than once). Exposure was coded as 1 if the respondent reported at least one occurrence and 0 otherwise. Violence victimization was defined as experiencing at least one of the following events, “Someone pulled a knife or gun on you”; “Someone shot you” and “Someone cut or stabbed you.” Each item used the same response categories (0 = never, 1 = at least once, 2 = more than once), and exposure was coded as 1 if any of the events occurred at least once and 0 otherwise. Parental death was coded as 1 if either of biological parents had died before the respondent reached age of 18. Foster care was coded as 1 if the respondent reported ever having lived in a foster home (0 = no, 1 = yes). Material hardship was measured using a single parent-reported item indicating whether they had enough money to pay their bills (0 = no, 1 = yes).

### Parenting Satisfaction and Parenting Stress

The outcomes were parenting satisfaction and parenting stress at WIV. Parenting satisfaction was evaluated based on the degree to which parents agreed or disagreed with the statement, “I am happy in my role as parent.” Response categories include “strongly agree,” “agree,” “neither agree nor disagree,” “disagree,” and “strongly disagree”. Consistent with prior research (Henderson et al., 2016), parenting satisfaction was dichotomized given the highly skewed distribution of responses (74.6% “strongly agree” and 2.6% “disagree/strongly disagree”). Although dichotomization may reduce variability, the highly skewed distribution means that treating the variable as continuous would provide limited additional meaningful information, as most responses are concentrated in a single category. Also, although this assessment was skewed, it was consistent with other satisfaction measures such as the General Social Survey’s marital happiness (HAPMAR) measure, in which only 2 to 4% of the participants responded “not too happy” in any given year, compared to the high percentage who responded “very happy” or “pretty happy” (Henderson et al., 2016).

Parenting stress was determined by the extent to which parents agreed or disagreed with the statements, “The major source of stress in my life is my child(ren).” and “I feel overwhelmed by the responsibility of being a parent.” The response options were the same as parenting satisfaction. Based on Henderson et al. (2016), parenting stress was coded as 1 if participants responded “strongly agree” or “agree” to either statement, it was coded as 0.Similarly, the relatively low endorsement of higher-stress responses (12%–14%) limited variability across categories, making dichotomization a practical approach to capture the presence of high parenting stress.

### Analytic Plan

Descriptives analyses of ACEs and parenting satisfaction and parenting stress were conducted by SPSS (IBM Corp, 2023). Then we used Latent Gold (Vermunt & Magidson, 2021) to conduct LCA and the Bolck-Croon-Hagenaars (BCH) approach to examining distal outcomes (Bolck et al., 2004) to explore how patterns of parental ACEs predict parenting satisfaction and parenting stress. No additional covariates were included in the models.

The BCH approach is a latent class analysis technique that involves three steps. First, we identified latent class memberships of 15 ACEs in mothers and fathers separately. To identify the most appropriate number of classes, we ran multiple LCA models starting with 1-class solution and then incrementally increased the number of classes. Model selection was guided by statistical fit criteria, such as Akaike Information Criterion (AIC), Bayesian Information Criterion (BIC), Vuong-Lo-Mendell-Rubin (VLMR) likelihood Ratio Test and entropy (Nylund-Gibson & Choi, 2018). A better model solution was determined by lower AIC and BIC, and higher entropy. The VLMR test compares the fit of a k-class model with k-1 class model. If the p-value < 0.05, it indicates that adding an extra class significantly improves model fit. As we evaluated the latent class models, we not only relied on model fit statistics but also prioritized the significance of the classes from a theoretical perspective and the extent to which they represent participants in our study (Hipp & Bauer, 2006; Nylund et al., 2007; Shin et al., 2018). After selecting the most appropriate model, we saved the model classification posterior probabilities as predictors. We then applied BCH step 3 to examine the association between maternal and paternal ACE class memberships and parenting satisfaction and stress.

## Results

### Descriptive Statistics of Key Study Variables

Table 1 provides descriptive statistics for ACEs, parenting satisfaction and parenting stress. Among the 15 ACEs, the most common ACEs were parental separation (26.6%) and parental alcohol abuse (25.0%), followed by emotional abuse (16.7%), violence victimization (16.7%), material hardship (16.0%), parental incarceration (14.7%) and witnessing violence (14.3%). The prevalence of all other ACEs was below 10%. The sample consists of more mothers (60.6%) than fathers (39.4%). The majority of parents identified as White (59.0%), with Black parents representing the second-largest racial group (25.2%). The average age of parents was 29.2 years (*SD = 1.7*). Most parents (74.6%) reported being satisfied with their role as a parent, while 20.4% found parenting stressful.

**Table 1:** Descriptive Statistics of ACEs and Parenting Satisfaction and Parenting Stress

Key Study Variables	Yes (*n*, %)/ M (SD)	Missing (*n*,%)	Mothers (*N* = 1562) Yes (*n*, %)/M (SD)	Fathers (*N* = 1016) Yes (*n*, %)/M (SD)
Physical abuse	158 (6.1%)	597 (23.1%)	97 (6.2%)	61 (6.0%)
Physical neglect	84 (3.3%)	592 (23.0%)	47(3.0%)	37 (3.6%)
Sexual abuse	94 (3.6%)	587 (22.8%)	65 (4.2%)	29 (2.9%)
Emotional abuse	431 (16.7%)	38 (1.5%)	296 (19.0%)	135 (13.3%)
Emotional neglect	212 (8.2%)	70 (2.7%)	148 (9.5%)	64 (6.3%)
Parental separation	685 (26.6%)	359 (13.9%)	419 (26.8%)	266 (26.2%)
Parental conflict	59 (2.3%)	967 (37.5%)	37 (2.4%)	22 (2.2%)
Parental incarceration	378 (14.7%)	3 (0.1%)	231 (14.8%)	147 (14.5%)
Parental alcohol abuse	644 (25.0%)	385 (14.9%)	386 (24.7%)	258 (25.4%)
Household suicidal attempt	135 (5.2%)	24 (0.9%)	93 (6.0%)	42 (4.1%)
Witnessing violence	368 (14.3%)	16 (0.6%)	196 (12.5%)	172 (16.9%)
Violence victimization	431 (16.7%)	10 (0.4%)	165 (10.6%)	266 (26.2%)
Parental death	99 (3.8%)	501 (19.4%)	58 (3.7%)	41 (4.0%)
Foster care	45 (1.7%)	504 (19.5%)	31 (2.0%)	14 (1.4%)
Material hardship	413 (16.0%)	408 (15.8%)	255 (16.3%)	158 (15.6%)
Parenting satisfaction	1925 (74.6%)	0	1220 (78.1%)	704 (69.3%)
Parenting stress	526 (20.4%)	0	332 (21.3%)	193 (19.0%)
Hispanic/Latino	285 (11.1%)		177 (11.3%)	108 (10.6%)
White	1522 (59.0%)		913 (58.5%)	609 (59.9%)
Black	650 (25.2%)		393 (25.25%)	257 (25.3%)
Asian	43 (1.7%)		27 (1.7%)	16 (1.6%)
Other	60 (2.3%)		40 (2.6%)	20 (2.0%)
Age	29.19 (1.72)	0	29.05 (1.71)	29.39 (1.72)

### Latent Class Analysis of Maternal and Paternal ACEs

Fit statistics of LCA for maternal and paternal ACEs were presented in Tables 2 and 3. For both maternal and paternal ACEs, models with 1 to 10 classes were estimated. Model selection was based on a combination of statistical fit indices (e.g., BIC, AIC, VLMR), entropy, and the interpretability and substantive distinctiveness of the resulting classes.

**Table 2:** Fit Statistics for LCA Models of Maternal ACEs

Classes	BIC	AIC	L²	*p*	VLMR	*p*	Entropy
1	14047.75	13967.45	4195.44	< 0.001			1.00
2	13762.09	13596.12	3792.11	< 0.001	403.33	< 0.001	0.47
3	13782.29	13530.67	3694.66	< 0.001	97.45	< 0.001	0.49
4	13819.12	13481.83	3613.82	< 0.001	80.84	< 0.001	0.50
**5**	**13886.88**	**13463.94**	**3563.93**	**< 0.001**	**49.89**	**< 0.001**	**0.52**
6	13973.49	13464.88	3532.88	< 0.001	31.05	0.10	0.53
7	14062.87	13468.61	3504.60	< 0.001	28.28	0.14	0.53
8	14147.31	13467.38	3471.38	< 0.001	33.23	0.07	0.63
9	14225.06	13459.48	3431.47	< 0.001	39.90	0.02	0.67
10	14323.46	13472.22	3412.21	< 0.001	19.26	0.34	0.70

**Table 3:** Fit Statistics for LCA Models of Paternal ACEs

Classes	BIC	AIC	L²	*p*	VLMR	*p*	Entropy
1	9226.17	9152.32	3092.04	< 0.001			1.00
2	9047.54	8894.91	2802.64	< 0.001	289.40	< 0.001	0.57
3	9026.61	8795.20	2670.92	< 0.001	131.71	< 0.001	0.63
4	9083.62	8773.43	2617.16	< 0.001	53.76	< 0.001	0.62
5	**9152.59**	**8763.63**	**2575.35**	**< 0.001**	**41.81**	**< 0.001**	**0.66**
6	9227.18	8759.43	2539.15	< 0.001	36.20	0.05	0.72
7	9302.99	8756.47	2504.19	< 0.001	34.96	< 0.001	0.71
8	9382.35	8757.04	2472.77	< 0.001	31.43	0.11	0.67
9	9467.25	8763.17	2446.89	< 0.001	25.88	0.11	0.68
10	9551.99	8769.13	2420.85	< 0.001	26.04	0.21	0.68

For maternal ACEs, the BIC favored a 2-class solution, whereas the AIC favored a 5-class solution. The VLMR test indicated improved fit up to the 5-class model but not beyond. We selected the 5-class solution as the preferred model because it represented the most differentiated solution supported by the model comparison results and yielded substantively distinct classes. While the BIC favored more parsimonious solutions, these models primarily distinguished between low versus high levels of adversity and did not capture meaningful heterogeneity in ACEs patterns. In contrast, the 5-class solution differentiated distinct constellations of co-occurring adversities (e.g., violence, abuse, and household dysfunction), which were central to the research questions of the present study.

For paternal ACEs, the BIC suggested a 3-class solution, whereas the AIC favored models with more classes. The VLMR test indicated improved fit up to the 5-class solution, with the comparison between the 5- and 6-class models reaching only marginal significance (*p* = .05). Although the 6-class model showed slightly higher entropy, this improvement was modest and did not correspond to clearer or more substantively meaningful class distinctions. The 5-class solution was therefore retained as a balance between statistical fit and interpretability. Similar to the maternal results, more parsimonious models primarily reflected general levels of adversity, whereas the 5-class solution identified distinct and interpretable patterns of ACEs without introducing unnecessary complexity.

Entropy values suggested moderate classification certainty (maternal = 0.52; paternal = 0.66). These values indicate that class separation was not perfect, particularly for maternal ACEs, and that some overlap across classes remained. Accordingly, class membership and between-class comparisons should be interpreted with caution.

**Fig. 1:**
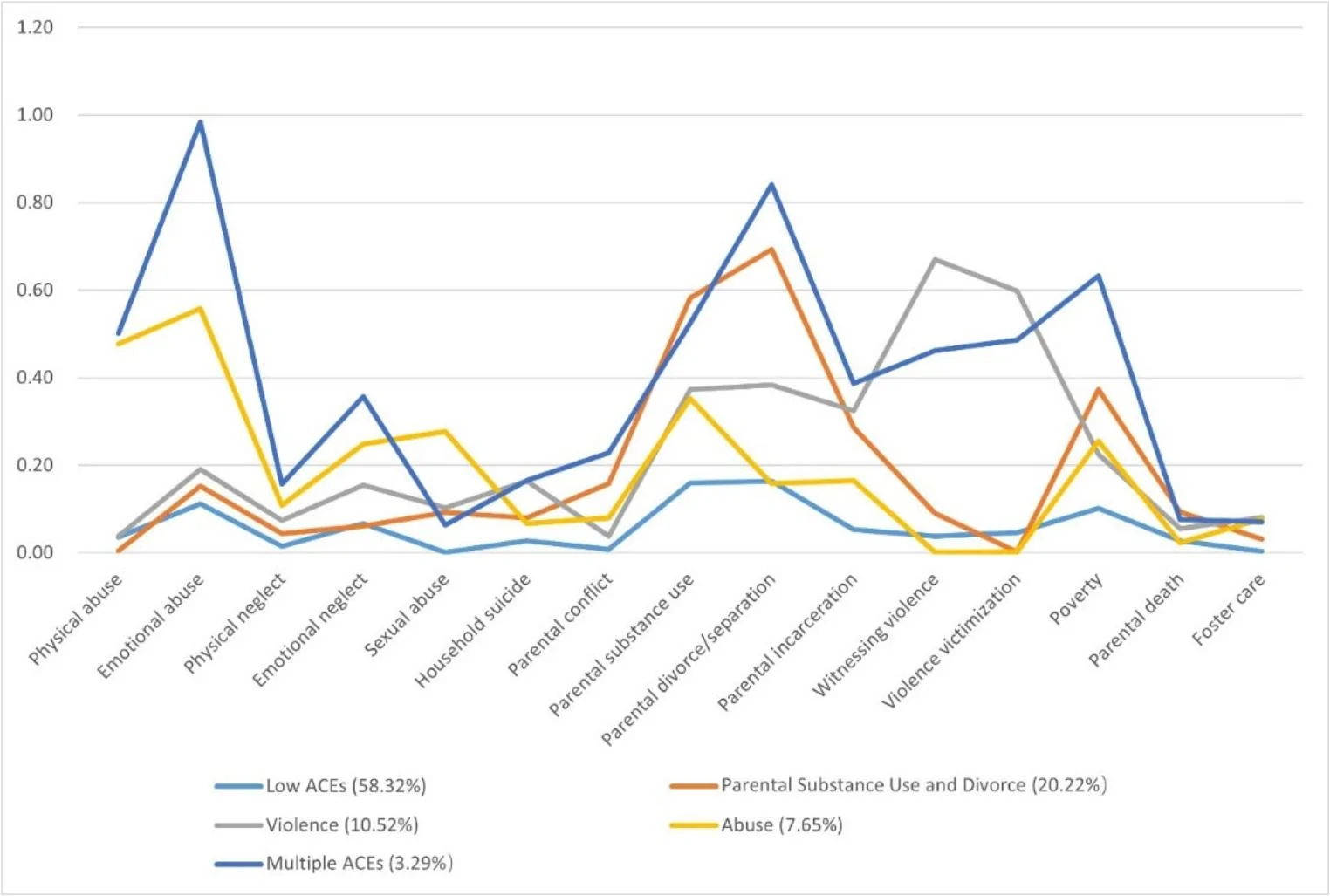
Probabilities of ACEs Indicators in Five Class Model for Mothers

Figure 1 shows the percentage of class size for 5-class models and item-response probabilities for maternal ACEs. The largest class was the Low ACEs class (58.32%), which was characterized by low probabilities of endorsing any of the 15 ACEs. Participants in the Parental Substance Use and Divorce class (20.22%) had high probability of experiencing parental substance use problems (0.58), and parental divorce/separation (0.69). The Violence class (10.52%) was marked by higher probabilities of witnessing violence (0.67), being a victim of violence (0.60). The Abuse class (7.65%) was characterized by high probabilities of abuse-related ACEs, including physical abuse (0.48), sexual abuse (0.28), and emotional abuse (0.56). The Multiple ACEs class (3.29%) was characterized by higher probabilities of experiencing different dimensions of ACEs, including household dysfunction such as parental substance abuse (0.52) and parental divorce/separation (0.84); childhood maltreatment such as physical abuse (0.50) and emotional abuse (0.98), and environmental ACEs such as poverty (0.63), witnessing violence (0.46) and violence victimization (0.49).

**Fig. 2:**
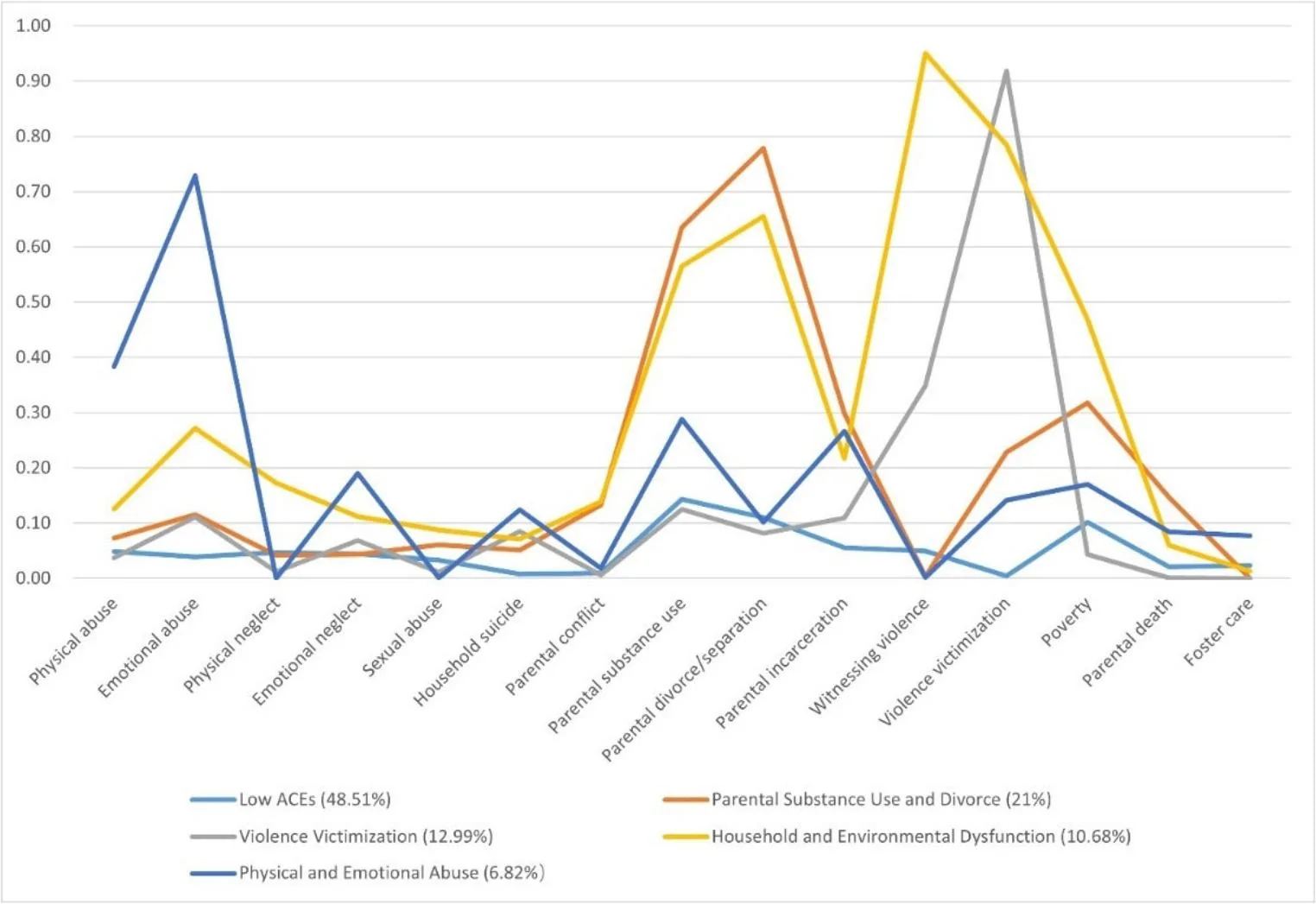
Probabilities of ACEs Indicators in Five Class Model for Fathers

The percentage of class size for 5-class models and item-response probabilities for paternal ACEs was presented in Fig. 2. The majority of fathers fell into the Low ACEs class (48.51%). The second largest class was the Parental Substance Use and Divorce class (21%) by exhibiting high probability of endorsing parental substance use problems (0.64) and divorce (0.78). The Violence Victimization class (12.99%) was marked by high probability of being a victim of violence (0.92). The Household and Environmental Dysfunction class (10.68%) was characterized by higher probability of parental substance use (0.57), parental divorce (0.66), witnessing violence (0.95), being a victim of violence (0.78), and living in poverty (0.47). The Physical and Emotional Abuse class (6.82%) was differentiated from other classes by exhibiting relatively high probability of experiencing physical abuse (0.38) and emotional abuse (0.73).

### ACEs Latent Classes and Parenting Satisfaction

Next, we used the BCH approach to examine the association between ACEs class memberships and parenting satisfaction for both mothers and fathers. The results of class membership-weighted logistic regression analyses were presented in Table 4. For mothers, significant association was observed in ACEs class memberships and parenting satisfaction. Paired comparison test indicates that all ACEs classes were significantly associated with lower likelihood of parenting satisfaction except for the Parental Substance Use and Divorce class. Mothers in the Violence class had 48% lower odds (*OR* = 0.52) of reporting high parenting satisfaction, 68% lower odds (*OR* = 0.32) for the Abuse class, and 60% lower odds (*OR* = 0.40) for the Multiple ACEs class. In addition, compared to the Parental Substance Use class, the Abuse class was less likely to report being satisfied as a parent.

**Table 4:** Odds Ratio from Class Membership-weighted Logistic Regression Analyses

	Parenting Satisfaction				Parenting Stress		
	OR	Wald	Post-hoc	*p*	OR	Wald	*p*
**Intercept**	4.69	175.34		< 0.001	0.25	169.92	< 0.001
**Class Memberships (Mothers)**		16.52		< 0.001		6.05	0.20
Class 2: Parental Substance Use and Divorce	0.77				1.05		
Class 3: Violence	0.52		3 < 1		0.99		
Class 4: Abuse	0.32		4 < 1, 4 < 2		1.66		
Class 5: Multiple ACEs	0.40		5 < 1		2.32		
**Intercept**	3.03	77.89		< 0.001	0.21	122.62	< 0.001
**Class Memberships (Fathers)**		11.04		0.03		1.37	0.85
Class 2: Parental Substance Use and Divorce	0.66				1.24		
Class 3: Violence Victimization	0.66				1.18		
Class 4: Household and Environmental Dysfunction	0.55		4 < 1		1.34		
Class 5: Physical and Emotional Abuse	0.34		5 < 1		0.96		

Note. Reference class = Class 1: Low ACEs

For fathers, class memberships were also significantly associated with parenting satisfaction. After further examination of paired comparison, only the Household and Environmental Dysfunction class (45% lower odds) and the Physical and Emotional Abuse class (66% lower odds) differed from the Low ACEs class in the likelihood of parenting satisfaction.

### ACEs Latent Classes and Parenting Stress

Similar to parenting satisfaction, we also used the BCH approach to examine the association between ACEs class memberships and parenting stress for both mothers and fathers. The results of class membership weighted-logistic regression analyses were also shown in Table 4. For both mothers and fathers, ACEs class memberships were not significantly associated with parenting stress at the omnibus level. Pairwise comparisons indicated that the Multiple ACEs class was more likely to report parenting stress than the Low ACEs class; however, this difference.

should be interpreted cautiously given the non-significant omnibus test.

## Discussion

Although previous research has established the link between maternal ACEs scores and parenting stress (Lange et al., 2019; Steele et al., 2016), much less attention has been given to the role of paternal ACEs or to the positive aspects of parenting, such as parenting satisfaction. Further, researchers rarely examined different patterns of ACEs influencing parenting stress and parenting satisfaction in mothers and fathers separately. To address these gaps, the present study used latent class analysis (LCA) to explore how different patterns of maternal and paternal ACEs affect parenting satisfaction and parenting stress.

The first aim was to use LCA to identify the ACEs class for both mothers and fathers. Consistent with our hypothesis, we uncovered slightly different ACEs class structures between mothers and fathers, despite both groups exhibiting five classes. For mothers, the five classes were: Low ACEs, Parental Substance Use and Divorce, Violence, Abuse, and Multiple ACEs. For fathers, the five classes are: Low ACEs, Parental Substance Use and Divorce, Violence Victimization, Household and Environmental Dysfunction, and Physical and Emotional Abuse. The most important distinction is that maternal ACEs structure showed two abuse-related classes: the Multiple ACEs class and the Abuse class, while paternal ACEs structure only revealed one abuse class. Also, maternal ACEs structure showed a class including sexual abuse. For mothers, the Violence class included traumatic events of witnessing violence and experiencing violence victimization, whereas for fathers, the Violence Victimization class was primarily characterized by violence victimization alone. These findings suggest that mothers and fathers may experience and internalize childhood adversities in distinct ways. Specifically, the observed gender differences in exposure to abuse- and violence-related experiences may reflect gendered patterns of vulnerability to early relational trauma and external threats, with lasting implications for their parenting perceptions and behaviors.

Despite the original ACEs measure’s emphasis on family-related adversities, we identified a distinct class characterized by high probabilities of exposure to violence-related ACEs in both maternal and paternal ACEs patterns. Consistent with a prior study (Lee et al., 2020), exposure to community violence, whether it’s witnessing or being a victim is an important defining characteristic of ACEs classes. These findings underscore the importance of incorporating community violence into parenting research, as such patterns of adversity may have important implications for parenting experiences.

Overall, both mothers and fathers exposed to different class memberships of ACEs had lower likelihood of parenting satisfaction than mothers and fathers having no exposure to any type of ACEs. The findings suggest that early adversity may impair parents’ ability to derive fulfillment and meaning from their parenting role, even when accounting for the specific patterns of ACEs exposure. This aligns with attachment theory, which posits that insecure early attachments may diminish individuals’ parenting experiences via negative internal working model (Hinnen et al., 2009). In contrast, although prior studies have found that higher cumulative ACEs exposure is associated with greater parenting stress (Lange et al., 2019; Steele et al., 2016), the present study did not find a significant association between ACEs patterns and parenting stress. Although a pairwise comparison suggested that individuals in the Multiple ACEs class reported higher parenting stress than those in the Low ACEs class for mothers, this difference should be interpreted with caution given the non-significant omnibus test, and may reflect a localized contrast rather than a robust overall association. One possible explanation is that parenting stress may be more sensitive to the overall accumulation of adversity rather than specific configurations of ACEs; however, this interpretation remains speculative, as the present study did not directly compare cumulative and class-based approaches. Future research directly comparing cumulative risk and latent class approaches to ACEs would help clarify their relative implications for parenting stress. Additionally, parenting stress may be more strongly influenced by contemporaneous factors such as economic hardship, relationship strain or child-related challenges, which may attenuate the effects of early adversity. It is also possible that broader psychological stressors mediate the association between ACEs and parenting-specific stress. Furthermore, the limited measurement of parenting stress and its dichotomization in the current study may have constrained our ability to detect associations with ACEs patterns.

These findings underscore the importance of examining both positive and negative aspects of parenting. First, parenting stress and parenting satisfaction reflect different dimensions of parenting processes rather than opposite ends of a single continuum. Parenting stress reflects strain associated with the demands of the parenting role and has been linked to harsher or more reactive parenting behaviors (Jackson & Choi, 2018; Niu et al., 2018). In contrast, parenting satisfaction reflects the degree to which parents experience fulfillment and meaning in their parenting role and has been associated with more adaptive parenting processes, such as lower levels of harsh discipline and more positive parent–child relationships (Carpenter & Donohue, 2006; Rogers & Matthews, 2004).

Second, the findings suggest that early adversity may not primarily increase parenting-related strain, but may instead undermine the positive and rewarding aspects of parenting. This pattern would not be evident if only negative parenting outcomes were examined. Examining both dimensions therefore provides a more comprehensive understanding of how early adversity may shape parenting and, in turn, child development.

For both mothers and fathers, the Parental Substance Use and Divorce class did not differ significantly from the Low ACEs class in parenting satisfaction. This is somewhat unexpected considering that prior research has linked parental substance use to child physical and emotional abuse (Freisthler & Kepple, 2019; Kepple, 2018), which were associated with lower likelihood of parenting satisfaction in the current study for both mothers and fathers. One possible explanation is that, within the latent class framework, the effects of parental substance use and divorce may be attenuated when considered alongside other co-occurring adversities, making their associations less detectable compared to findings from prior research. Additionally, parenting satisfaction reflects a subjective evaluation of the parenting role, which may not directly correspond to earlier exposure to specific types of adversity.

One notable gender difference was that the Violence class in mothers was associated with a lower likelihood of parenting satisfaction compared to the Low ACEs class, whereas fathers in the Violence Victimization class did not differ significantly from the Low ACEs class. Gender differences in the association between violence exposure and parenting satisfaction may reflect differences in how such experiences are interpreted and carried into later parenting. For example, prior research suggests that women may be more likely to develop interdependent self-construals that emphasize emotional connection (Cross & Madson, 1997), and may therefore be more sensitive to disruptions in relational trust and security. In contrast, men may be more likely to develop independent self-construals and process adverse experiences in ways that are less directly tied to relational expectations. However, these mechanisms were not directly examined in the present study and should be interpreted as speculative. Alternative explanations should also be considered. For instance, the observed gender differences may reflect differences in how violence exposure is recalled or reported, with women potentially more likely to report interpersonal and relational aspects of adversity that are more closely tied to parenting satisfaction. In addition, the observed gender differences may be specific to this cohort. Participants in Add Health came of age in the late 1990s and early 2000s, and the ways in which violence was experienced, interpreted, and shaped later life experiences may differ by gender within this historical context. However, the present study was not designed to examine these processes directly, and the mechanisms underlying these gender differences remain unclear.

Although ACEs constellations differed between mothers and fathers, the overall findings for parenting outcomes showed both convergence and divergence across domains. Specifically, ACEs patterns were not significantly associated with parenting stress for either mothers or fathers at the omnibus level. In contrast, ACEs patterns were associated with lower parenting satisfaction in both groups, although the specific classes and magnitude of associations differed. Parenting stress may be more strongly shaped by current contextual demands, such as economic strain, relationship quality, and child characteristics, which may attenuate the effects of early adversity similarly for both mothers and fathers. For parenting satisfaction, although maternal and paternal ACEs classes were distinct in their composition, they showed partial overlap in key dimensions of adversity, such as household dysfunction, abuse, and violence exposure. These shared dimensions may help explain why different constellations of ACEs were associated with similar patterns of parenting satisfaction across groups, even though the specific class structures differed. Importantly, maternal and paternal ACEs classes were not directly comparable in their composition; therefore, convergence should be interpreted at the level of overall patterns rather than one-to-one correspondence between specific classes.

### Limitations and Strengths

Several limitations should be acknowledged. First, parenting satisfaction was measured using a single item, and parenting stress was assessed with two items and subsequently dichotomized, which may have reduced variability and statistical power and limited the ability to detect associations. For example, coding “agree” responses for parenting satisfaction as 0 may overlook meaningful variation in parents’ positive experiences and reduce the sensitivity of the measure. However, these variables have been utilized in previous research, and the large national sample may mitigate the limitation (Henderson et al., 2016).

Another limitation concerns the retrospective nature of the ACEs measures. ACEs were assessed using retrospective reports across multiple waves, including WI (ages 12–18), WIII (ages 18–26), and WIV (ages 24–32). In particular, at WIII and WIV, participants were asked to recall ACEs that occurred before the 6th grade or before age 18. Such retrospective reporting in adulthood may be subject to recall bias, as individuals may not accurately retrieve or reconstruct past experiences (Reuben et al., 2016). In addition, adult participants may be reluctant to report sensitive experiences due to concerns about distress or embarrassment (Reuben et al., 2016). Such biases may affect the accuracy of ACEs indicators and, in turn, influence latent class membership by introducing misclassification or reducing the distinctiveness between classes. Furthermore, the relatively low entropy value suggests less precise differentiation between classes, which may also reflect measurement limitations in capturing heterogeneous patterns of adversity.

The models also did not include additional contextual covariates, such as current mental health or family structure, which are known to influence parenting experiences. Although aspects of socioeconomic adversity were partially captured through ACEs indicators (e.g., material hardship), these measures do not fully reflect individuals’ current socioeconomic conditions. As a result, the observed associations between ACEs patterns and parenting outcomes may still be subject to residual confounding. Future research incorporating more comprehensive contextual factors would help clarify these relationships.

In addition, we used data from Add Health that focuses on a specific age group of parents (25 to 34 years old). While this age range is relevant, parenting satisfaction and stress are dynamic across the lifespan of parenthood. For instance, parental age may influence levels of stress and satisfaction, particularly among teenager parents. Additionally, the study could not determine the specific age at which participants transitioned into parenthood, which was a notable limitation given that the brain undergoes significant changes during this period for both mothers and fathers (Kim et al., 2010, 2014). Future research should consider these developmental transitions to provide a more comprehensive understanding of parenting experiences.

Finally, although the present study focused on parenting stress and parenting satisfaction as outcomes, future research may extend this work by examining whether parenting experiences serve as mechanisms linking distinct ACEs profiles to subsequent parent and child health outcomes. Such research may further advance understanding of the intergenerational consequences of ACEs and inform family-based prevention and intervention efforts.

Despite the limitations, we contributed to the field by using a large national sample to identify distinct patterns of ACEs for mothers and fathers and demonstrating that specific combinations of ACEs that were associated with lower parenting satisfaction in both mothers and fathers. This finding indicates that the impact of ACEs on parenting varies based on the type of ACEs, parent gender, and parenting dimensions. Finally, most ACEs literature rarely gives attention to ACEs intergenerational effects (Wang et al., 2024), our findings on how ACEs influence parenting experiences can enhance professionals’ awareness, including those in child protective services, pediatrics, family therapists, and professionals for parenting education. Integrating this knowledge into their services has the potential to disrupt the cycle of ACEs transmission and promote healthier family outcomes.

### Conclusion and Implications

Our study advances the field by revealing different patterns of ACEs in fathers and mothers, as well as how different ACEs patterns may differentially influence fathers’ and mothers’ parenting experiences. This gender-responsive and trauma-informed approach provides significant implications for intervention, program and policy design to break the cycle of intergenerational trauma.

First, when supporting families, child welfare and mental health professionals can consider administering ACEs screening for parents to better understand the context of parenting. Professionals may pay special attention to the sexual abuse experience that mothers experience to see whether there is any unresolved sexual trauma that manifests in their parenting behavior. If so, timely referral pathways to specialized trauma treatment should be provided.

As the majority of the ACEs literature has focused almost exclusively on mothers, our findings on fathers’ distinct ACEs patterns and their associations with paternal parenting behaviors underscore a need to direct research efforts and programmatic resources toward fathers. Given that fathers are as likely as mothers to experience ACEs and that paternal involvement in childrearing has grown substantially in recent decades, fatherhood programs, child welfare systems, and family court practices can adopt gender-responsive, trauma-informed approaches that acknowledge fathers’ trauma histories and support fathering, instead of penalizing and criticizing father absence, to ultimately promote children’s long-term wellbeing.

Finally, our findings that violence-related ACEs significantly influence parenting, with a stronger impact on maternal parenting, highlight the need for ACEs prevention and intervention to extend beyond the family unit to include structural and community-level action. It is inspiring that CDC plays an active role in ACEs prevention, while unfortunately the widely used CDC-Kaiser ACEs scale and its related ACEs surveillance system (e.g., Behavioral Risk Factor Surveillance System) does not capture community violence, which may profoundly constrain the scope of policy and programmatic responses. We therefore advocate for a more comprehensive ACEs screening and surveillance system at the government level, as well as sustained investment in community-level violence program to support families, and ultimately break the intergenerational cycle of ACEs.

## Data Availability

This study used public-use data from the National Longitudinal Study of Adolescent to Adult Health (Add Health). The dataset can be accessed through the Inter-university Consortium for Political and Social Research (ICPSR) at the following link: [https://www.icpsr.umich.edu/web/ICPSR/studies/21600](https:/www.icpsr.umich.edu/web/ICPSR/studies/21600).
